# Chiral Optical Properties of Möbius Graphene Nanostrips

**DOI:** 10.1021/acs.jpclett.3c00925

**Published:** 2023-05-04

**Authors:** Marina E. Razzhivina, Ivan D. Rukhlenko, Nikita V. Tepliakov

**Affiliations:** † Information Optical Technologies Center, ITMO University, Saint Petersburg 197101, Russia; ‡ School of Physics, Institute of Photonics and Optical Science, The University of Sydney, Camperdown, NSW 2006, Australia; ¶ Department of Materials and The Thomas Young Centre for Theory and Simulation of Materials, Imperial College London, London SW7 2AZ, United Kingdom

## Abstract

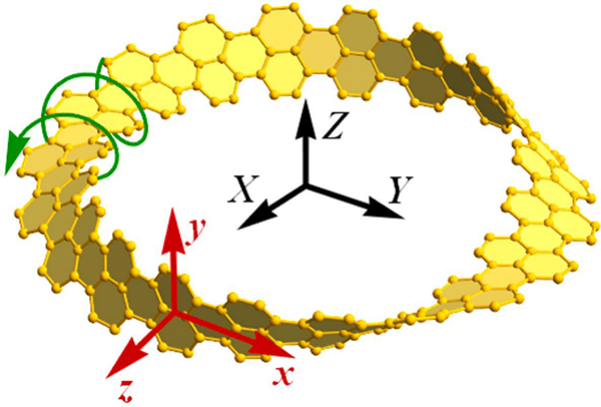

The advancement of optical technology demands the development of chiral nanostructures with a strong dissymmetry of optical response. Here, we comprehensively analyze the chiral optical properties of circular twisted graphene nanostrips, with a particular emphasis on the case of a Möbius graphene nanostrip. We use the method of coordinate transformation to analytically model the electronic structure and optical spectra of the nanostrips, while employing the cyclic boundary conditions to account for their topology. It is found that the dissymmetry factors of twisted graphene nanostrips can reach 0.01, exceeding the typical dissymmetry factors of small chiral molecules by 1–2 orders of magnitude. The results of this work thus demonstrate that twisted graphene nanostrips of Möbius and similar geometries are highly promising nanostructures for chiral optical applications.

Chiral nanostructures, characterized by the lack of mirror symmetry, are used as catalysts for chiral synthesis,^1−4^ circularly polarized light emitters,^5−7^ sensors of chiral organic molecules,^8−11^ and spintronics logic devices.^12−15^ Many of these applications require developing new chiral nanostructures with enhanced optical response. A particularly promising avenue in this regard is the nanostructures based on graphene, as they combine graphene’s unique electronic properties with size quantization of charge carriers.^16−18^ Examples of chiral graphene nanostructures include twisted monolayer graphene quantum dots,^19,20^ bilayer graphene quantum dots with mutual rotation between layers,^21−24^ twisted graphene nanoribbons,^25−27^ and chiral carbon nanotubes.^28−30^ These nanostructures were shown to possess strong chiroptical responses in the visible, ultraviolet, and near-infrared spectral regions.

Recently, Segawa et al. synthesized a new chiral graphene nanostructure: graphene nanobelts in the form of a Möbius strip.^31^ Such nanostructures are synthesized in the bottom-up fashion starting from nanoribbons made of carbon, bromine, and hydrogen and functionalized with oxygen-containing groups.^32^ The geometry of these nanoribbons forces them to twist along their axis and simultaneously bend, forming a loop. Given a right length of the nanoribbon, its opposite ends are rotated by 180° with respect to each other, thus resulting in a Möbius nanostrip. The spectroscopic studies revealed strong optical activity of the fabricated Möbius graphene nanostrips, with the dissymmetry factors ranging between 10^–3^ and 10^–2^. The shape of the Möbius nanostrip is not only chiral but also topologically nontrivial due to it being a nonorientable surface.^33^ This feature was shown to give rise to topologically protected edge states and the quantum spin Hall effect in Möbius structures.^34,35^ Notably, Möbius graphene nanostrips with zigzag edges also feature a spin-polarized ground state,^36^ which is ferromagnetic (unlike in plain nanoribbons) because such nanostrips have effectively a single edge.^37^ It is natural to wonder how the topologically nontrivial geometry of Möbius graphene nanostrips affects their optical activity, which has not been assessed to date.

In this Letter, we comprehensively analyze the optical activity of Möbius graphene nanostrips. In order to account for the nontrivial geometry of the Möbius strip, we introduce a curvilinear space in which a twisted nanostrip transforms into a plain graphene nanoribbon characterized by modified cyclic boundary conditions. Absorption of circularly polarized radiation by the nanostrip is described in the curvilinear coordinates using a topologically transformed light–matter interaction operator. We establish the selection rules of optically active transitions in twisted graphene nanostrips and confirm that the Möbius nanostrip indeed features circular dichroism due to its chiral shape. We further analyze twisted graphene nanostrips of other dimensions and geometries in search of the structures with the strongest dissymmetry of optical absorption. It is found that the dissymmetry factors of twisted graphene nanostrips can reach values ∼0.01, which makes these chiral nanostructures a highly attractive material for chiral nanophotonics.

Consider a plane graphene nanoribbon of length *L*, which is made of atoms arranged in the armchair configuration, and denote the maximum number of atoms across the nanoribbon’s width as *n*. Figure 1a shows a plane nanoribbon with *n* = 5 and the orientation of the Cartesian axes (*x*, *y*, *z*) associated with its surface. A twisted circular nanostrip is obtained by twisting a plane nanoribbon *m* times along its *x*-axis and connecting the opposite ends. Without loss of generality, we focus on three types of twisted nanoribbons shown in Figure 1b: an achiral circular nanostrip with *m* = 0, a Möbius nanostrip with *m* = 1, and a doubly twisted nanostrip with *m* = 2. The Cartesian coordinates (*X*, *Y*, *Z*) of atoms in a twisted circular nanostrip are expressed through the Cartesian coordinates (*x*, *y*, *z*) of atoms in the plane nanoribbon as
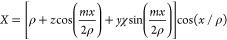
1
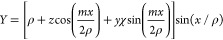
2
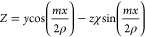
3where ρ = *L*/(2π) is the radius of the circular nanostrip and χ = ±1 distinguishes between the twist directions.

**Figure 1:**
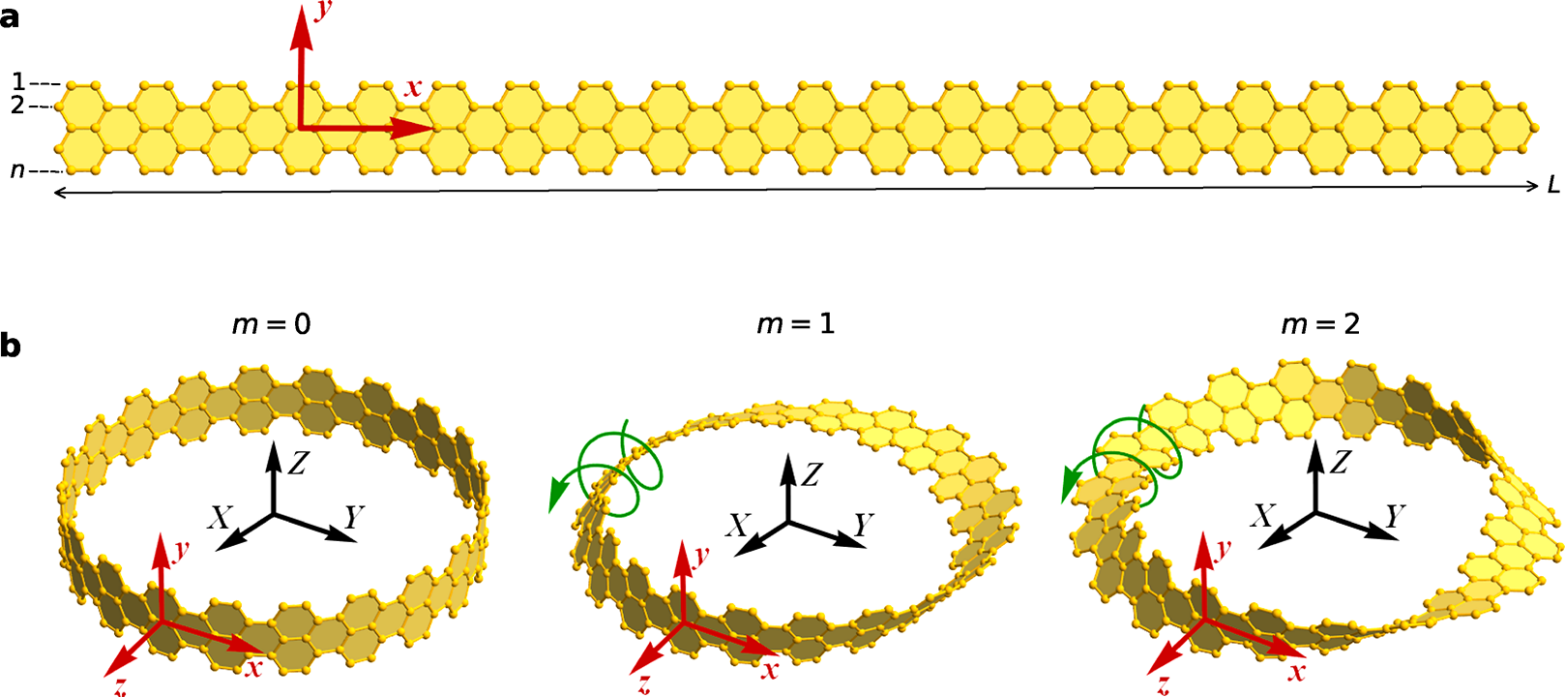
(a) Plane graphene nanoribbon of length *L* and with *n* atoms across the width and (b) circular graphene nanostrips obtained by twisting the nanoribbon *m* times around its *x* axis and connecting the opposite ends. The green spiral arrows indicate the direction of twist in the displayed enantiomers with χ = 1. The nanostrips are a plane nanoribbon in the local curvilinear coordinates (*x*, *y*, *z*), and the origin of Cartesian coordinates (*X*, *Y*, *Z*) is at the center of the circular nanostrip. In all cases, *n* = 5 and *L* = 85 nm.

The interaction of a twisted nanostrip with light can be conveniently described using the local coordinates (*x*, *y*, *z*), in which the nanostrip is plain and has simple electronic structure, yet the light–matter interaction is modified by the inverse coordinate transformation
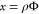
4
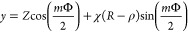
5
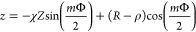
6where  and Φ = arctan(*Y*/*X*).

By solving the eigensystem problem for the standard tight-binding Hamiltonian of a graphene nanoribbon,^38,39^ written as a function of the local coordinates (*x*, *y*, *z*), we find the wave functions ψ_*μk*_ and energies *E*_*μk*_ of the *p*_*z*_ electrons. The wave functions are the Bloch waves of the form
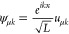
7where *u*_*μk*_ is the periodic Bloch amplitude, μ labels energy bands, and *k* is the electron wavenumber in the *x*-direction. This wavenumber determines the local velocity of electrons on the surface of the nanostrip as well as the projection of their angular momentum on the Cartesian *Z*-axis of the circular nanostrip as ⟨*μk*|*L*_*Z*_|*μk*⟩ = *ℏρk*.

The quantization of the electron wavenumber *k* (and angular momentum *L*_*Z*_, correspondingly) is determined by the continuity of the wave function at the connected ends of the nanoribbon. For even *m*, which describe the nanostrips of trivial topology, the cross section of the nanostrip is oriented the same way at the two ends, as demonstrated in Figure 2a. The corresponding boundary condition
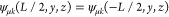
8yields the trivial quantization rule
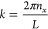
9or *L*_*Z*_ = *ℏn*_*x*_, where *n*_*x*_ ∈ *Z*. For odd *m*, corresponding to the nontrivial topology, the cross section of the nanostrip is rotated by *mπ* upon the twist, as shown in Figure 2b, and the same boundary condition reads
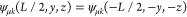
10

**Figure 2:**
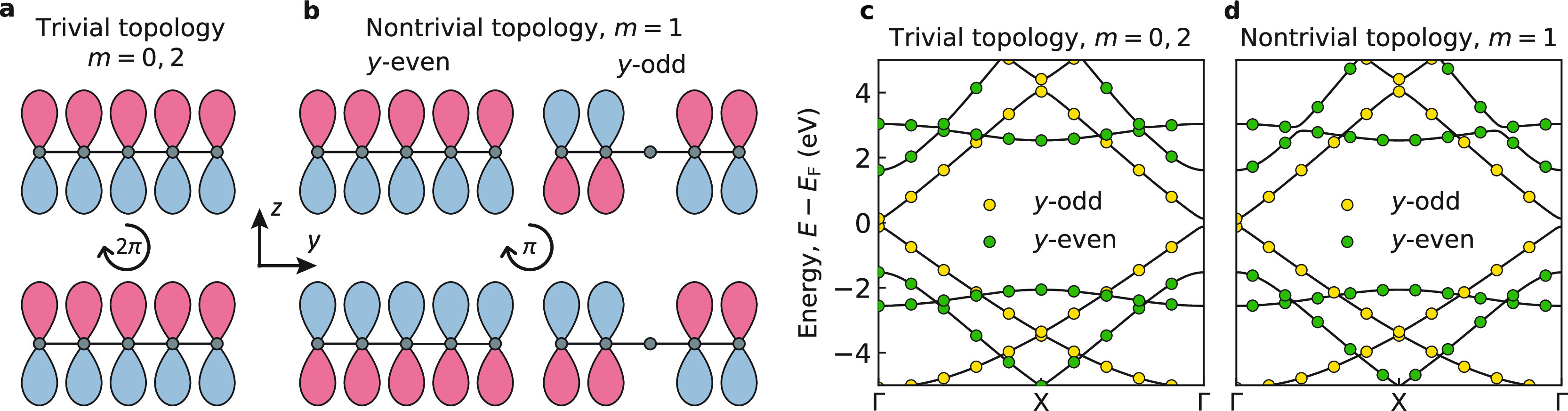
Rotation of the wave functions formed by *p*_*z*_-orbitals of carbon atoms in the cross section of a graphene nanoribbon with *n* = 5 upon the formation of a circular nanostrip with (a) trivial and (b) nontrivial topology. The cross section is rotated in the *yz*-plane either by 2π in the case of even number of twists (*m* = 0, 2) or by π in the case of odd number of twists (*m* = 1). Panel b shows states of different parities with respect to reflection *y* → −*y*. Energy bands of circular graphene nanostrips with (c) even and (d) odd number of twists. Yellow and green circles correspond to the states that are odd and even with respect to *y*, respectively.

Note that the Bloch amplitudes are odd functions of *z* because they originate from the *p*_*z*_-orbitals of carbon atoms, *u*_*μk*_(*y*, – *z*) = −*u*_*μk*_(*y*, *z*). Therefore, the quantization rule following from eq 10 depends on the parity of the Bloch amplitude with respect to *y*. One can see in Figure 2b that when *u*_*μk*_ is an odd function of *y*, the wave function is continuous at the connected ends of the nanostrip, *u*_*μk*_(−*y*, – *z*) = *u*_*μk*_(*y*, *z*), and the electron wavenumber *k* is quantized according to eq 9. In contrast, the wave function which is even with respect to *y* changes sign upon the rotation by *mπ*, *u*_*μk*_(−*y*, – *z*) = −*u*_*μk*_(*y*, *z*), and the quantization rule becomes
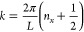
11or *L*_*Z*_ = *ℏ*(*n*_*x*_ + 1/2). We thus obtain that in Möbius graphene nanostrips, the *Z*-projection of angular momentum can assume semi-integer values of *ℏ*, contrary to the systems of trivial geometry.

Figure 2c,d compares the band structures of circular nanostrips with even and odd numbers of twists. One sees that different quantization rules of the electron wavenumber for even and odd Bloch amplitudes in a Möbius nanostrip shift the even energy states by π/*L* with respect to the odd states. This prevents vertical transitions between the bands of opposite parities, resulting in the red shift of the absorption spectrum noticeable for small *L*.

Absorption and circular dichroism (CD) spectra of randomly oriented circular graphene nanostrips are given by
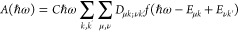
12
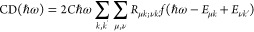
13where *C* = 32π^3^*N*_*c*_/(3ℏ*c*), *N*_*c*_ is the volumetric concentration of the nanostrips, the summation is performed over all occupied states |*μk*⟩ and all unoccupied states , *D*_*μk*;*νk*′_ = |⟨*μk*|**d**|*νk*′⟩|^2^ is the transition probability, *R*_*μk*;*νk*′_ = Im⟨*μk*|**d**|*νk*′⟩·⟨*νk*′|**m**|*μk*⟩ is the rotatory strength of the transition,^40,41^**d** and **m** are the electric and magnetic dipole moment operators, and *f*(*ℏω*) is the spectral line shape function.

The matrix elements of the dipole moments are expressed through the coordinate and momentum operators in the original Cartesian coordinates, **R** = (*X*, *Y*, *Z*) and **P** = −*iℏ*(∂_*X*_, ∂_*Y*_, ∂_*Z*_), as
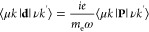
14
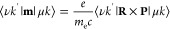
15Note that we do not include the topological contribution to the magnetic moment in eq 15. Such contribution is relevant for the Möbius graphene nanostrips with zigzag edges, whose bandstructures are spin-polarized,^36,42^ but not for armchair graphene nanostrips analyzed in this study. On the other hand, armchair-edge Möbius nanostrips made of materials other than graphene may potentially feature topologically protected states due to non-negligible spin–orbit coupling.^33^ Matrix elements of the dipole moments are evaluated in the Supporting Information through the conversion of **R** and **P** into the curvilinear coordinates (*x*, *y*, *z*) and subsequent integration of the modified operators over the Bloch states of the original flat nanoribbon. The result is presented in Table 1.

**Table 1:** Parameters *D*_*μk*;*νk*′_ and *R*_*μk*;*νk*′_ for Circular Graphene Nanostrips with *m* = 0, 1, 2^a^

	*m* = 0		*m* = 1		*m* = 2	
*k*′ – *k*	*D*_*μk*;*νk*′_	*R*_*μk*;*νk*′_	*D*_*μk*;*νk*′_	*R*_*μk*;*νk*′_	*D*_*μk*;*νk*′_	*R*_*μk*;*νk*′_
0	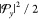	0	–	–	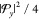	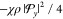
±π/*L*	–	–	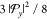	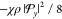	–	–
±2π/*L*	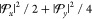	0	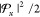	0	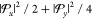	0
±3π/*L*	–	–	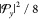	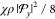	–	–
±4π/*L*	–	–	–	–	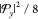	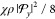

a (ξ = *x*, *y*) is the momentum matrix element calculated using the Bloch states of the nanoribbon; *D*_*μk*;*νk*′_ is in units  and *R*_*μk*;*νk*′_ is in units .

The selection rules of interband transitions in circular graphene nanostrips are seen to vary significantly depending on the number of the nanoribbon’s twists *m*. Absorption of light by circular nanostrips with *m* = 0 occurs via vertical transitions with Δ*k* = *k*′ – *k* = 0 (Δ*L*_*Z*_ = 0) and diagonal transitions with Δ*k* = ± 2π/*L* (Δ*L*_*Z*_ = ± *ℏ*). Möbius nanostrips with *m* = 1 absorb via six diagonal transitions accompanied by the transfer of momentum Δ*k* = ± π/*L*, ±2π/*L*, and ±3π/*L*, corresponding to Δ*L*_*Z*_ = ±*ℏ*/2, ±*ℏ*, and ±3*ℏ*/2. Finally, doubly twisted nanostrips with *m* = 2 absorb via vertical transitions with Δ*k* = 0 (Δ*L*_*Z*_ = 0) and diagonal transitions with Δ*k* = ±2π/*L* (Δ*L*_*Z*_ = ±*ℏ*) and ±4π/*L* (Δ*L*_*Z*_ = ±2*ℏ*). While absorption occurs upon both *x*- and *y*-polarized transitions, the optical activity is observed only for transitions which are polarized in the *y*-direction, because chirality of the nanostrip stems from its rotation in the cross section, i.e., the *yz*-plane in the local coordinates. This means that the CD signal is produced only by transitions between the bands of opposite parities.

The relative strength of the optical activity of circular graphene nanostrips can be conveniently characterized by the dissymmetry factor (*g*-factor) of optically active transitions^43^*g*_*μk*;*νk*′_ = 4*R*_*μk*;*νk*′_/*D*_*μk*;*νk*′_ and the *g*-factor spectrum *g*(*ℏω*) = 2CD(*ℏω*)/*A*(*ℏω*). The typical *g*-factors of small chiral molecules and semiconductor nanocrystals range between ±(10^–3^–10^–2^),^19,22,43−45^ while the maximum values of *g*-factor (±2) describe the situation when the system totally transmits one circular polarization. The data of Table 1 show that the *g*-factors of optically active transitions in circular graphene nanostrips scale linearly with the nanostrip’s radius as *g*_*μk*;*νk*′_ ∝ ± *qρ*, where *q* = ω/*c* is the wavenumber of light.

The intensities of the CD peaks and the peaks in the g-factor spectrum are limited by the finite widths of the absorption peaks and the decrease of the peaks’ separation with the nanostrip’s length. For example, the CD signal of Möbius nanostrips is generated via four transitions separated by Δ*k* = ±π/*L* and Δ*k* = ±3π/*L* (see Table 1). The respective pairs of the transitions have rotatory strengths that are equal in magnitudes and opposite in signs. The partial overlap of the adjacent absorption peaks yields the following the maximal value of the CD signal in the nanostrips:  ∼ , where 2γ is the full width at half-maximum (fwhm) of the peak. In graphene nanostructures *∂E*_*νk*_/*∂k* ∼ *ℏv*_F_, where *v*_F_ is the Fermi velocity, so that we finally get an estimate
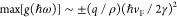
16Hence, while the strengths of individual optically active transitions grow linearly with the nanostrip’s size due to the linear growth of the dipole moment of the structure, the relative CD signal weakens as ∝1/ρ due to the increasing overlap of the absorption peaks. We cannot, however, assess the *g*-factors of chiral graphene nanostrips with very small ρ, since the coordinate transformation method used in this study requires the width of the nanostrip to be much smaller than its radius, |*y*| ≪ ρ.

Absorption spectra of Möbius (*m* = 1) and doubly twisted (*m* = 2) nanostrips with *n* = 5 and *L* = 85 nm are shown in Figure 3a. Owing to the large length and the resulting quasi-continuity of the energy spectra of the nanostrips, the difference between their absorption spectra is negligible and not reflected in the figure. The absorption is contributed by transitions polarized either along or perpendicular to the nanoribbon’s length. These contributions are shown by the yellow and green areas in the figure. One sees that the *x*-polarized transitions occur over the entire spectrum, whereas the *y*-polarized transitions occur only in the visible range and above. The absence of the *y*-polarized transitions in the infrared range is due to the fact that the two energy bands near the Fermi level have the same parities with respect to the *y* axis (see the band structure in Figure 2).

**Figure 3:**
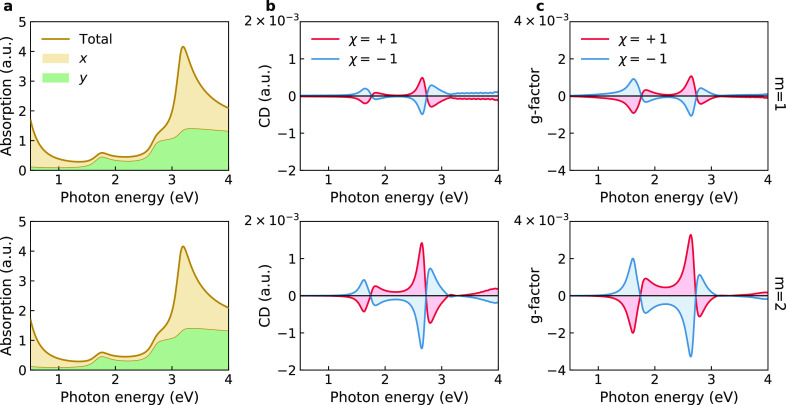
(a) Absorption, (b) CD, and (c) *g*-factor spectra of chiral graphene nanostrips with *m* = 1, 2, *n* = 5 atoms across the nanoribbon, and a length of *L* = 85 nm. The yellow and green areas under the absorption spectrum show relative contributions from the *x*- and *y*-polarized transitions; the colors of the CD spectra differentiate between the two enantiomers of the nanostrips (χ = ±1).

The CD spectra of the nanostrips are plotted in Figure 3b. Unlike absorption, the CD signal is seen to strongly depend on the number of twists. Note that the infrared CD signal is absent because only the *y*-polarized transitions in the nanostrips are optically active. The CD of the doubly twisted nanostrip is several times stronger than the CD of the Möbius nanostrip of the same size. Since the nanostrips absorb equally, this implies that the doubly twisted nanostrips exhibit larger dissymmetry of optical response and are thus more appealing for chiroptical applications. This is confirmed by the *g*-factor spectra of the chiral graphene nanostrips in Figure 3c. Absorption, CD, and dissymmetry factor spectra of chiral graphene nanoribbons with other values of *n* are shown in Figures S1–S5.

Figure 4 further illustrates the dependence of the *g*-factor spectrum on the parameters of chiral graphene nanostrips. Only nanoribbons with odd *n*, capable of forming closed Möbius nanostrips, are considered. Such nanoribbons possess mirror symmetry with respect to reflection *y* → −*y*, necessary to satisfy the boundary conditions ψ(*L*/2, *y*, *z*) = ψ(−*L*/2, −*y*, −*z*) at the connected ends of the nanostrip composed of an integer number of unit cells. The density plots in Figure 4 show the preferential absorption of left-circularly polarized light by red and right-circularly polarized light by blue. One sees that *g*(*ℏω*) can reach 10^–2^, which is close to the experimental value for Möbius graphene nanobelts^31^ and exceeds the typical values for small chiral molecules and quantum dots by 1–2 orders of magnitude.^19,46,47^ Chiral molecular cylinders, morphologically similar to circular graphene nanostrips, can feature even larger dissymmetry factors of the order of 10^–1^, though these values are highly sensitive to structural fluctuations.^48^ In agreement with the above theoretical prediction, *g*(*ℏω*) in Figure 4 decreases like ∝1/*L*. This suggests that smaller graphene nanoribbons are better for chiroptical applications. Confirming this, Möbius graphene nanobelts with unusually large dissymmetry factors, fabricated by Segawa et al., were indeed characterized by the radius of only a few nanometers.^31^

**Figure 4:**
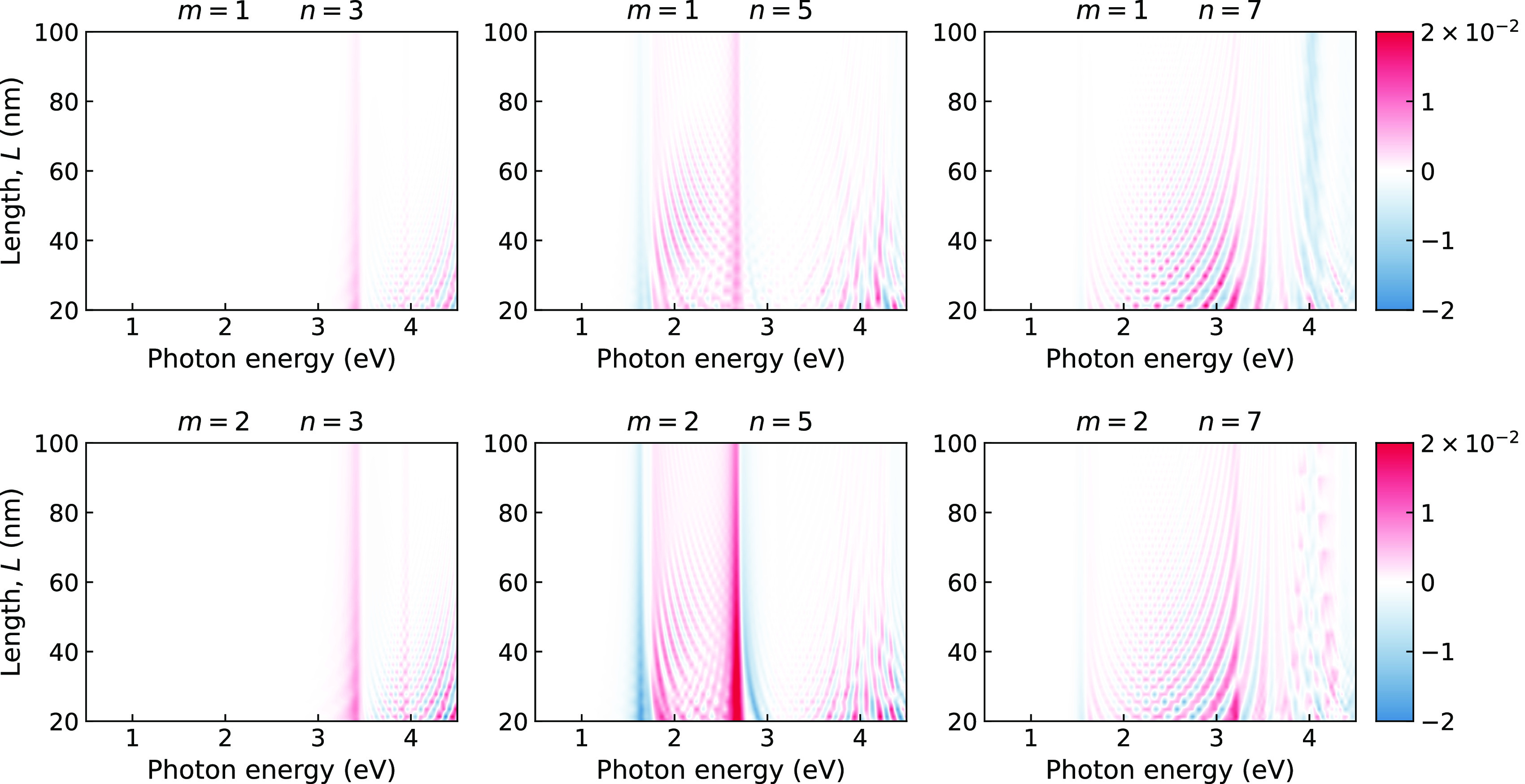
Dissymmetry factor spectra of chiral graphene nanostrips vs nanoribbon length *L* for *m* = 1, 2, *n* = 3, 5, 7, and χ = 1.

The two narrower graphene nanostrips with *n* = 3 and *n* = 5 feature pronounced CD peaks, the spectral positions of which do not change with the length of the nanostrip. For *n* = 3 there is a strong signal at 3.4 eV and a weaker signal of the same sign at 3.9 eV, whereas for *n* = 5 there are two equally pronounced peaks of different signs at 1.7 and 2.7 eV. The dissymmetry factors of the doubly twisted nanostrips are seen to be notably larger than those of the Möbius nanostrips of the same dimensions. The difference between *m* = 1 and *m* = 2 is the least prominent for the widest nanoribbons with *n* = 7, yet there is more interference in the optical spectra, with two weak peaks of opposite signs identifiable at 1.5 and 3.2 eV. To investigate the effect of larger widths on chiral optical properties of graphene nanostrips, in Figure S6 we show dissymmetry factors for *n* = 9, 11, and 13. This figure reveals similar trends in the optical spectra of wider graphene nanostrips, including relatively large *g*-factors of the order of 0.01. Overall, our results demonstrate that short twisted graphene nanostrips with *n* = 5 exhibit the strongest dissymmetry of optical response. This may be attributed to the fact that the armchair graphene nanoribbons with *n* = 3*p* + 2 (*p* = 1, 2, 3, ...) generally have richer electronic properties than other members of this family of carbon nanostructures.^38^

In future studies, the analytical model developed in this work can be extended to include various additional atoms, such as oxygen, to provide more accurate modeling of circular graphene nanostrips synthesized from organic precursors. Our theoretical approach can be also employed to analyze Möbius graphene nanostrips with zigzag edges, whose nontrivial topological and magnetic properties can have significant implications for their chiral optical response. Finally, the coordinate transformation method can potentially describe Möbius nanostrips made of other two-dimensional semiconductors, e.g., transition metal dichalcogenides or hexagonal boron nitride, thus opening up new perspectives for studying the influence of nontrivial topology on different materials.

To conclude, we have modeled optical activity of Möbius graphene nanostrips and similar graphene nanostructures with a different number of twists. In order to calculate electronic properties of these chiral nanostructures, we developed a coordinate transformation that turns a twisted graphene nanostrip into a plane graphene nanoribbon, whose electronic properties are obtained using the tight-binding Hamiltonian for *p*_*z*_-electrons. Upon the coordinate transformation, the chirality of the nanostrip is transferred to the curvature of the space, modifying the light–matter interaction operator. The nontrivial topology of the Möbius nanostrip is accounted for by the cyclic boundary conditions which distinguish between the energy bands of different parities. Further analytical calculations revealed that circular dichroism in twisted graphene nanostrips can only be observed upon transitions polarized across the nanostrip, whereas absorption is formed by the transitions polarized both across and along the axis of the nanoribbon. We further analyzed the dependence of the dissymmetry factor on the length and width of the Möbius and doubly twisted nanostrips. Among the analyzed structures, doubly twisted nanostrips of width *n* = 5 and smaller lengths were found to feature the strongest dissymmetry of optical response. Overall, the dissymmetry factors of small twisted graphene nanostrips can reach values ∼0.01, highlighting Möbius graphene nanostrips as highly promising nanostructures for applications in chiral nanophotonics.

## Supporting Information Available

Analytical calculations of the matrix elements of electric and magnetic dipole moments in curvilinear coordinates; Figures S1–S6 showing absorption, CD, and dissymmetry factor spectra of chiral graphene nanostrips with *n* = 3, 7, 9, 11, 13 (PDF)
Transparent Peer Review report available (PDF)
