# Supplementary material for: Chiral Optical Properties of Möbius Graphene Nanostrips
Source: J Phys Chem Lett. 2023 May 4;14(19):4426–32. doi: 10.1021/acs.jpclett.3c00925 (PMC10201574; doi:10.1021/acs.jpclett.3c00925)
Supplement: Supplementary file 1 — jz3c00925_si_001.pdf [file jz3c00925_si_001.pdf]

Supplementary Information for Chiral Optical Properties of Möbius Graphene Nanostrips

Marina E. Razzhivina,[†] Ivan D. Rukhlenko,[†,‡] and Nikita V. Tepliakov[*,¶]

†*Information Optical Technologies Center, ITMO University, Saint Petersburg 197101, Russia*

‡*School of Physics, Institute of Photonics and Optical Science, The University of Sydney, Camperdown, NSW 2006, Australia*

¶*Department of Materials and The Thomas Young Centre for Theory and Simulation of Materials, Imperial College London, London SW7 2AZ, United Kingdom*

E-mail: n.tepliakov20@imperial.ac.uk

Matrix elements of dipole moments

In order to calculate the matrix elements of the electric and magnetic dipole moments, we convert the momentum operator $\mathbf{P} = P_X\mathbf{e}_X + P_Y\mathbf{e}_Y + P_Z\mathbf{e}_Z$ in the original Cartesian coordinates (X, Y, Z) into the local curvilinear coordinates (x, y, z) of the nanostrip as

$$P_X = \frac{\partial x}{\partial X}p_x + \frac{\partial y}{\partial X}p_y + \frac{\partial z}{\partial X}p_z, \tag{1}$$

$$P_Y = \frac{\partial x}{\partial Y}p_x + \frac{\partial y}{\partial Y}p_y + \frac{\partial z}{\partial Y}p_z, \tag{2}$$

$$P_Z = \frac{\partial x}{\partial Z}p_x + \frac{\partial y}{\partial Z}p_y + \frac{\partial z}{\partial Z}p_z. \tag{3}$$

The result is give by

$$P_X = -\sin\left(\frac{x}{\rho}\right)p_x + \chi\cos\left(\frac{x}{\rho}\right)\sin\left(\frac{mx}{2\rho}\right)p_y, \tag{4}$$

$$P_Y = \cos\left(\frac{x}{\rho}\right)p_x + \chi\sin\left(\frac{x}{\rho}\right)\sin\left(\frac{mx}{2\rho}\right)p_y, \tag{5}$$

$$P_Z = \cos\left(\frac{mx}{2\rho}\right)p_y, \tag{6}$$

where it has been considered that the width of the nanoribbon is much smaller than its radius, $|y| \ll \rho$, and that the nanoribbon is locally flat, so that $z = 0$ and $p_z = 0$.

Similarly, the angular momentum in the original coordinates, $\mathbf{L} = \mathbf{R} \times \mathbf{P}$, is expressed through the curvilinear coordinates as

$$L_X = p_y\rho\cos\left(\frac{mx}{2\rho}\right)\sin\left(\frac{x}{\rho}\right), \tag{7}$$

$$L_Y = -p_y\rho\cos\left(\frac{mx}{2\rho}\right)\cos\left(\frac{x}{\rho}\right), \tag{8}$$

$$L_Z = \rho p_x. \tag{9}$$

We next need to calculate the interband matrix elements of the forms $f(x)p_x$ and $f(x)p_y$. Following the standard approach of the $\mathbf{k}\cdot\mathbf{p}$-perturbation theory, we calculate the matrix element of the momentum operators p_x and p_y using the periodic Bloch amplitudes $u_{\mu k}$ and $u_{\nu k'}$ and evaluate the matrix element of the slowly varying functions $f(x)$ by integrating them over the exponential envelopes e^{ikx}. The resulting matrix elements of the momentum operator are given by

$$\begin{aligned}\langle\mu k|P_X|\nu k'\rangle = &-\frac{i}{2}\langle\mu k|p_x|\nu k'\rangle\left(\delta_{k',k+\frac{2\pi}{L}} - \delta_{k',k-\frac{2\pi}{L}}\right)\\ &+\frac{i\chi\langle\mu k|p_y|\nu k'\rangle}{4}\left(\delta_{k',k+\frac{(m+2)\pi}{L}} + \delta_{k',k+\frac{(m-2)\pi}{L}} - \delta_{k',k-\frac{(m-2)\pi}{L}} - \delta_{k',k-\frac{(m+2)\pi}{L}}\right),\end{aligned} \tag{10}$$

$$\langle\mu k|P_Y|\nu k'\rangle = \frac{\langle\mu k|p_x|\nu k'\rangle}{2}\left(\delta_{k',k+\frac{2\pi}{L}} + \delta_{k',k-\frac{2\pi}{L}}\right)$$
$$- \frac{\chi\langle\mu k|p_y|\nu k'\rangle}{4}\left(\delta_{k',k+\frac{(m+2)\pi}{L}} - \delta_{k',k+\frac{(m-2)\pi}{L}} - \delta_{k',k-\frac{(m-2)\pi}{L}} + \delta_{k',k-\frac{(m+2)\pi}{L}}\right), \quad (11)$$

$$\langle\mu k|P_Z|\nu k'\rangle = \frac{\langle\mu k|p_y|\nu k'\rangle}{2}\left(\delta_{k',k+\frac{m\pi}{L}} + \delta_{k',k-\frac{m\pi}{L}}\right). \quad (12)$$

Similarly, the matrix elements of the angular momentum are given by

$$\langle\nu k'|L_X|\mu k\rangle = \frac{\rho\langle\nu k'|p_y|\mu k\rangle}{4i}\left(\delta_{k',k+\frac{(m+2)\pi}{L}} - \delta_{k',k+\frac{(m-2)\pi}{L}} + \delta_{k',k-\frac{(m-2)\pi}{L}} - \delta_{k',k-\frac{(m+2)\pi}{L}}\right), \quad (13)$$
$$\langle\nu k'|L_Y|\mu k\rangle = -\frac{\rho\langle\nu k'|p_y|\mu k\rangle}{4}\left(\delta_{k',k+\frac{(m+2)\pi}{L}} + \delta_{k',k+\frac{(m-2)\pi}{L}} + \delta_{k',k-\frac{(m-2)\pi}{L}} + \delta_{k',k-\frac{(m+2)\pi}{L}}\right), \quad (14)$$

$$\langle\nu k'|L_Z|\mu k\rangle = \rho\langle\nu k'|p_x|\mu k\rangle\delta_{k',k}. \quad (15)$$

Absorption and circular dichroism

The absorption probability and intensity of the CD signal are given by

$$|\langle \mu k|\mathbf{P}|\nu k'\rangle|^2 = \frac{|\langle \mu k|p_x|\nu k'\rangle|^2}{2}\left(\delta_{k',k+\frac{2\pi}{L}} + \delta_{k',k-\frac{2\pi}{L}}\right) + \frac{|\langle \mu k|p_y|\nu k'\rangle|^2}{8} \times \left(\delta_{k',k+\frac{(m+2)\pi}{L}} + \delta_{k',k+\frac{(m-2)\pi}{L}} + \delta_{k',k-\frac{(m-2)\pi}{L}} + \delta_{k',k-\frac{(m+2)\pi}{L}} + 2\delta_{k',k+\frac{m\pi}{L}} + 2\delta_{k',k-\frac{m\pi}{L}}\right), \quad (16)$$

$$\langle \mu k|\mathbf{P}|\nu k'\rangle\langle \nu k'|\mathbf{L}|\mu k\rangle = \chi\frac{\rho|\langle \mu k|p_y|\nu k'\rangle|^2}{8} \times \left(\delta_{k',k+\frac{(m+2)\pi}{L}} - \delta_{k',k+\frac{(m-2)\pi}{L}} - \delta_{k',k-\frac{(m-2)\pi}{L}} + \delta_{k',k-\frac{(m+2)\pi}{L}}\right). \quad (17)$$

Specifically, for $m = 0$

$$|\langle \mu k|\mathbf{P}|\nu k'\rangle|^2 = \frac{|\langle \mu k|p_x|\nu k'\rangle|^2}{2}\left(\delta_{k',k+\frac{2\pi}{L}} + \delta_{k',k-\frac{2\pi}{L}}\right) + \frac{|\langle \mu k|p_y|\nu k'\rangle|^2}{4}\left(\delta_{k',k+\frac{2\pi}{L}} + 2\delta_{k',k} + \delta_{k',k-\frac{2\pi}{L}}\right), \quad (18)$$

$$\langle \mu k|\mathbf{P}|\nu k'\rangle\langle \nu k'|\mathbf{L}|\mu k\rangle = 0. \quad (19)$$

For $m = 1$

$$|\langle \mu k|\mathbf{P}|\nu k'\rangle|^2 = \frac{|\langle \mu k|p_x|\nu k'\rangle|^2}{2}\left(\delta_{k',k+\frac{2\pi}{L}} + \delta_{k',k-\frac{2\pi}{L}}\right) + \frac{|\langle \mu k|p_y|\nu k'\rangle|^2}{8}\left(\delta_{k',k+\frac{3\pi}{L}} + 3\delta_{k',k+\frac{\pi}{L}} + 3\delta_{k',k-\frac{\pi}{L}} + \delta_{k',k-\frac{3\pi}{L}}\right), \quad (20)$$

$$\langle \mu k|\mathbf{P}|\nu k'\rangle\langle \nu k'|\mathbf{L}|\mu k\rangle = \chi\frac{\rho|\langle \mu k|p_y|\nu k'\rangle|^2}{8}\left(\delta_{k',k+\frac{3\pi}{L}} - \delta_{k',k+\frac{\pi}{L}} - \delta_{k',k-\frac{\pi}{L}} + \delta_{k',k-\frac{3\pi}{L}}\right). \quad (21)$$

Finally, for $m=2$

$$|\langle \mu k|\mathbf{P}|\nu k'\rangle|^2 = \frac{|\langle \mu k|p_x|\nu k'\rangle|^2}{2}\left(\delta_{k',k+\frac{2\pi}{L}} + \delta_{k',k-\frac{2\pi}{L}}\right) + \frac{|\langle \mu k|p_y|\nu k'\rangle|^2}{8}\left(\delta_{k',k+\frac{4\pi}{L}} + 2\delta_{k',k+\frac{2\pi}{L}} + 2\delta_{k',k} + 2\delta_{k',k-\frac{2\pi}{L}} + \delta_{k',k-\frac{4\pi}{L}}\right), \quad (22)$$

$$\langle \mu k|\mathbf{P}|\nu k'\rangle\langle \nu k'|\mathbf{L}|\mu k\rangle = \chi\frac{\rho|\langle \mu k|p_y|\nu k'\rangle|^2}{8}\left(\delta_{k',k+\frac{4\pi}{L}} - 2\delta_{k',k} + \delta_{k',k-\frac{4\pi}{L}}\right). \quad (23)$$

Bloch matrix elements

The momentum matrix elements calculated using Bloch amplitudes, $\langle \nu k' | \mathbf{p} | \mu k \rangle$, are obtained from the matrix representation of the tight-binding Hamiltonian, H. First, the momentum operator is calculated in the basis of sublattices as

$$\mathbf{p} = \frac{m_{\mathrm{e}}}{\hbar} \frac{\partial H}{\partial \mathbf{k}}. \tag{24}$$

Then the interband matrix elements of the momentum operator are calculated as

$$\langle \nu k' | \mathbf{p} | \mu k \rangle = \sum_{i,j} c^{*}_{\nu k' i} p_{ij} c_{\mu k j} \tag{25}$$

where $c_{\mu k j}$ is the jth component of the eigenvector corresponding to state $|\mu k\rangle$.

Optical spectra of chiral graphene nanostrips

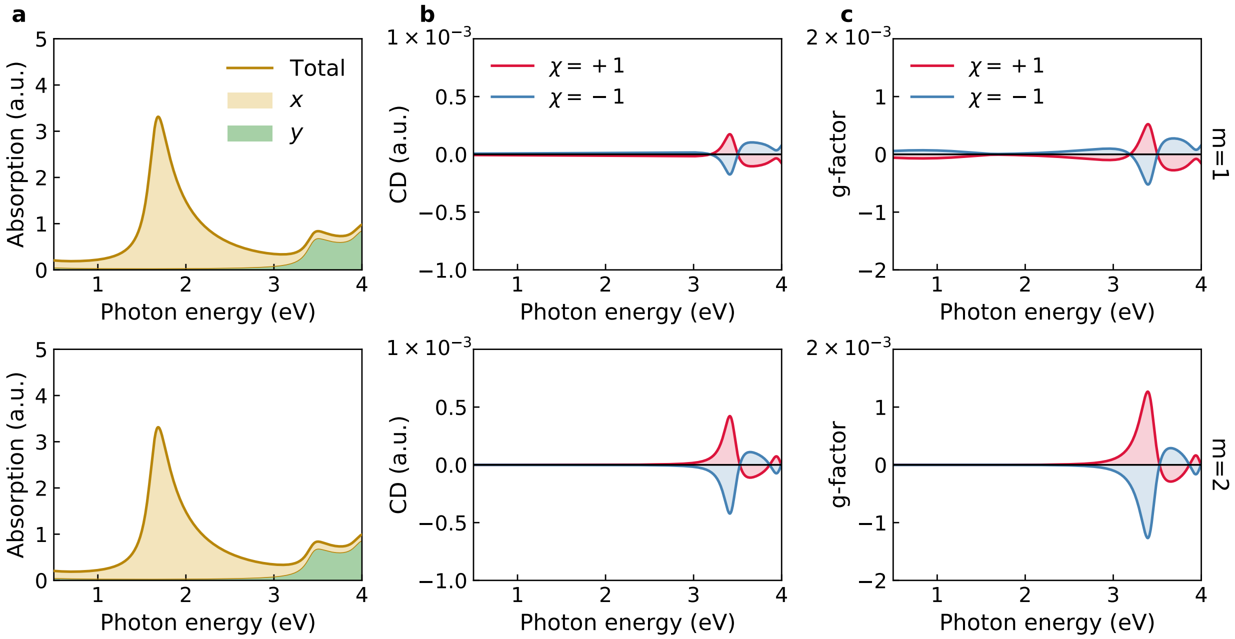

Figure S1: (a) Absorption, (b) CD, and (c) g-factor spectra of chiral graphene nanostrips with $m = 1, 2$, $n = 3$, and $L = 85$ nm. The yellow and green areas under the absorption spectrum show relative contributions from the x- and y-polarized transitions; the colors of the CD spectra differentiate between the two enantiomers of the nanostrips ($\chi = \pm 1$).

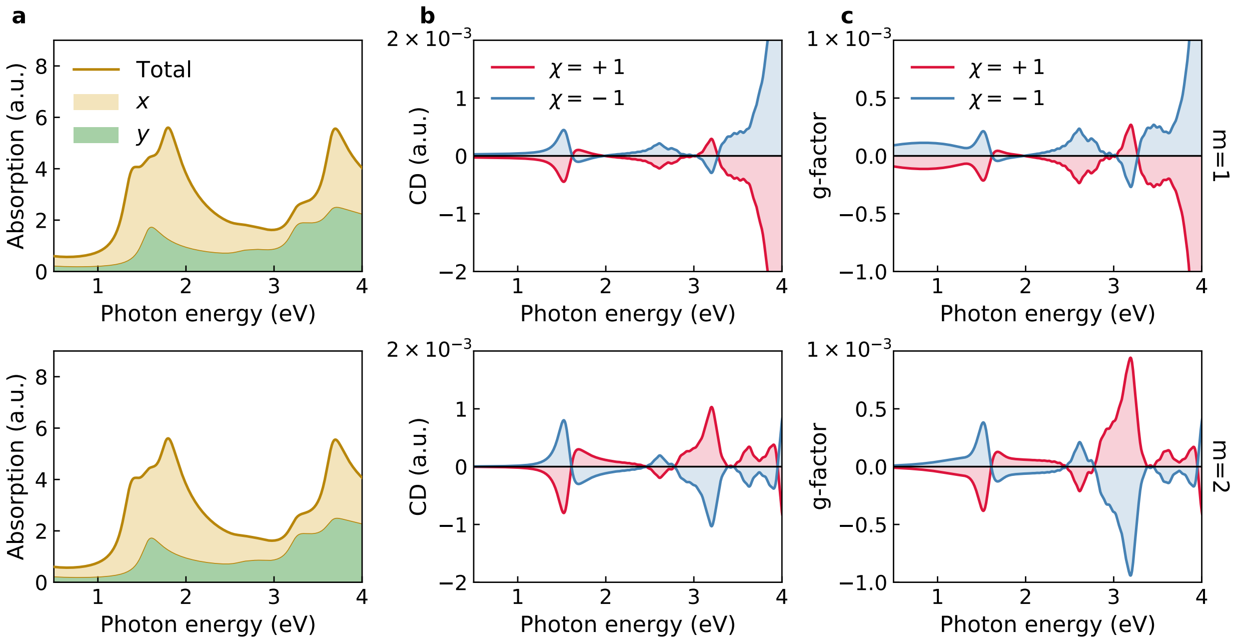

Figure S2: (a) Absorption, (b) CD, and (c) g-factor spectra of chiral graphene nanostrips with $m = 1, 2$, $n = 7$, and $L = 85$ nm. The yellow and green areas under the absorption spectrum show relative contributions from the x- and y-polarized transitions; the colors of the CD spectra differentiate between the two enantiomers of the nanostrips ($\chi = \pm 1$).

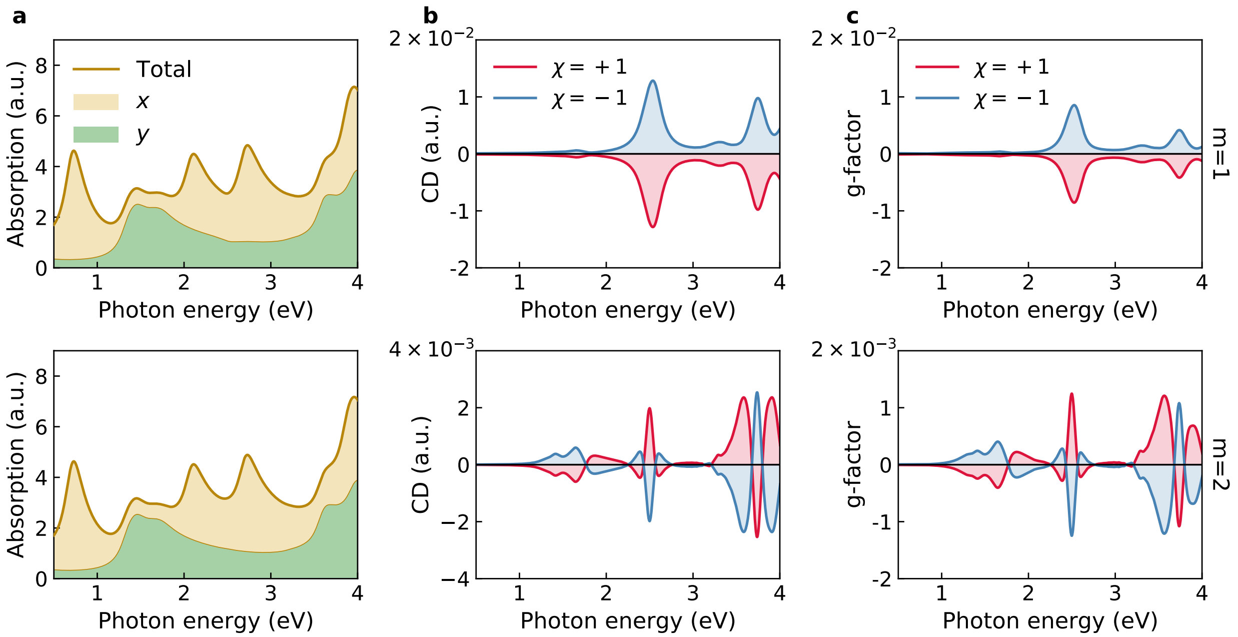

Figure S3: (a) Absorption, (b) CD, and (c) g-factor spectra of chiral graphene nanostrips with $m = 1, 2$, $n = 9$, and $L = 85$ nm. The yellow and green areas under the absorption spectrum show relative contributions from the x- and y-polarized transitions; the colors of the CD spectra differentiate between the two enantiomers of the nanostrips ($\chi = \pm 1$).

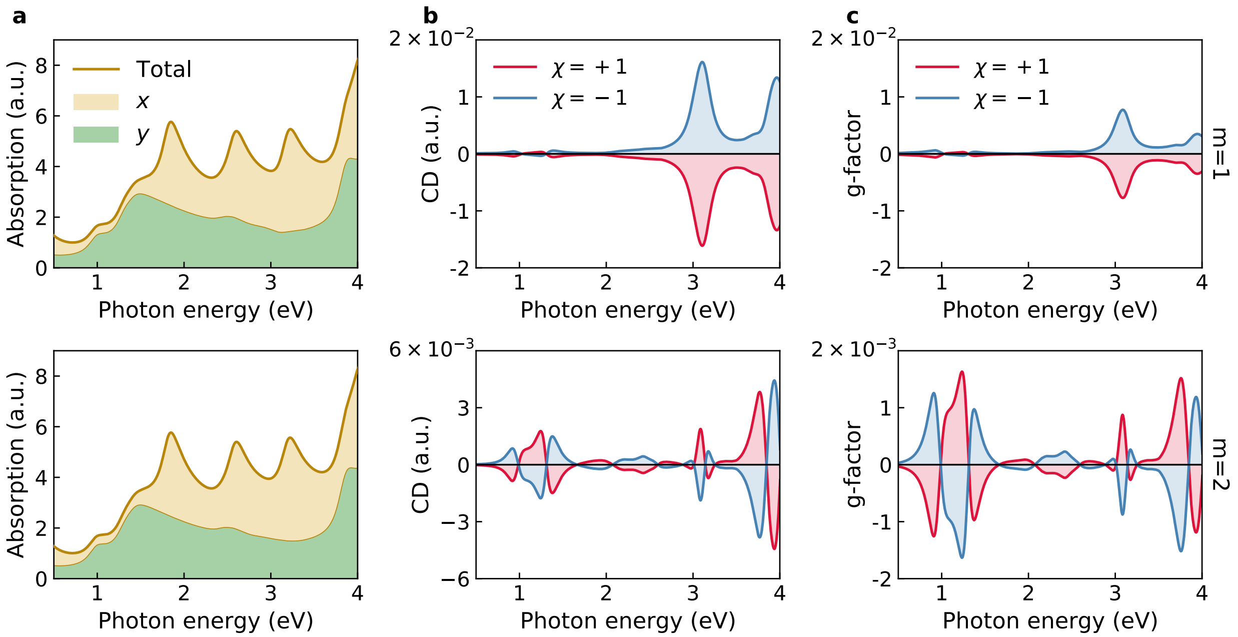

Figure S4: (a) Absorption, (b) CD, and (c) g-factor spectra of chiral graphene nanostrips with $m = 1, 2$, $n = 11$, and $L = 85$ nm. The yellow and green areas under the absorption spectrum show relative contributions from the x- and y-polarized transitions; the colors of the CD spectra differentiate between the two enantiomers of the nanostrips ($\chi = \pm1$).

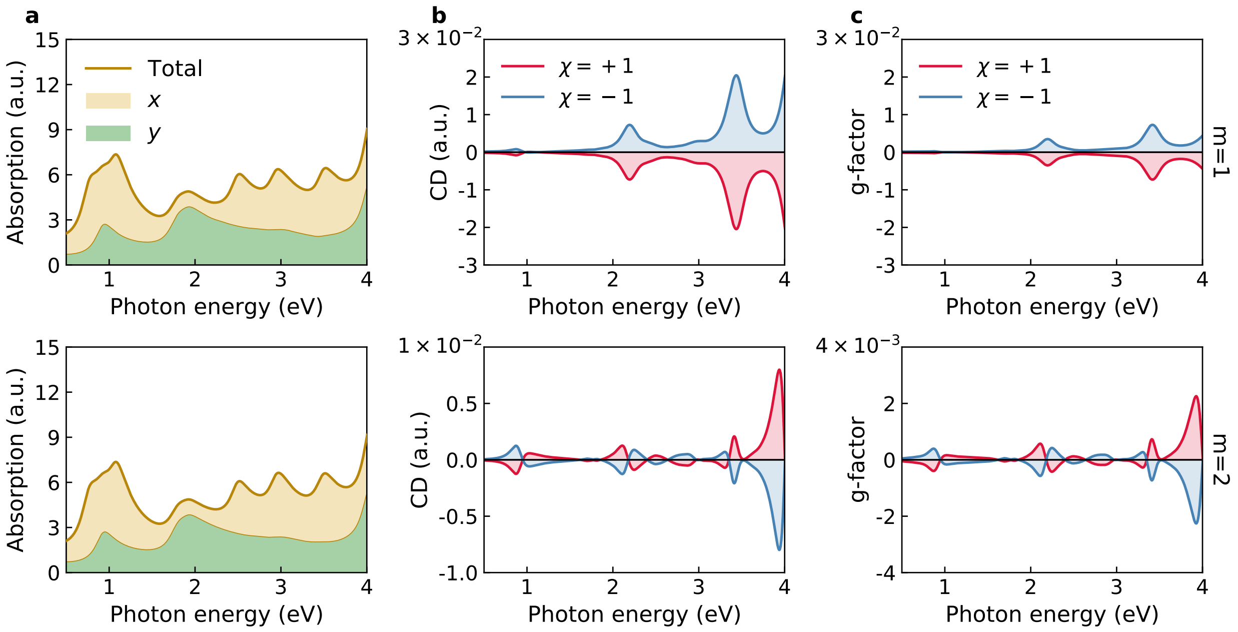

Figure S5: (a) Absorption, (b) CD, and (c) g-factor spectra of chiral graphene nanostrips with $m = 1, 2$, $n = 13$, and $L = 85$ nm. The yellow and green areas under the absorption spectrum show relative contributions from the x- and y-polarized transitions; the colors of the CD spectra differentiate between the two enantiomers of the nanostrips ($\chi = \pm 1$).

Dissymmetry factors of chiral graphene nanostrips

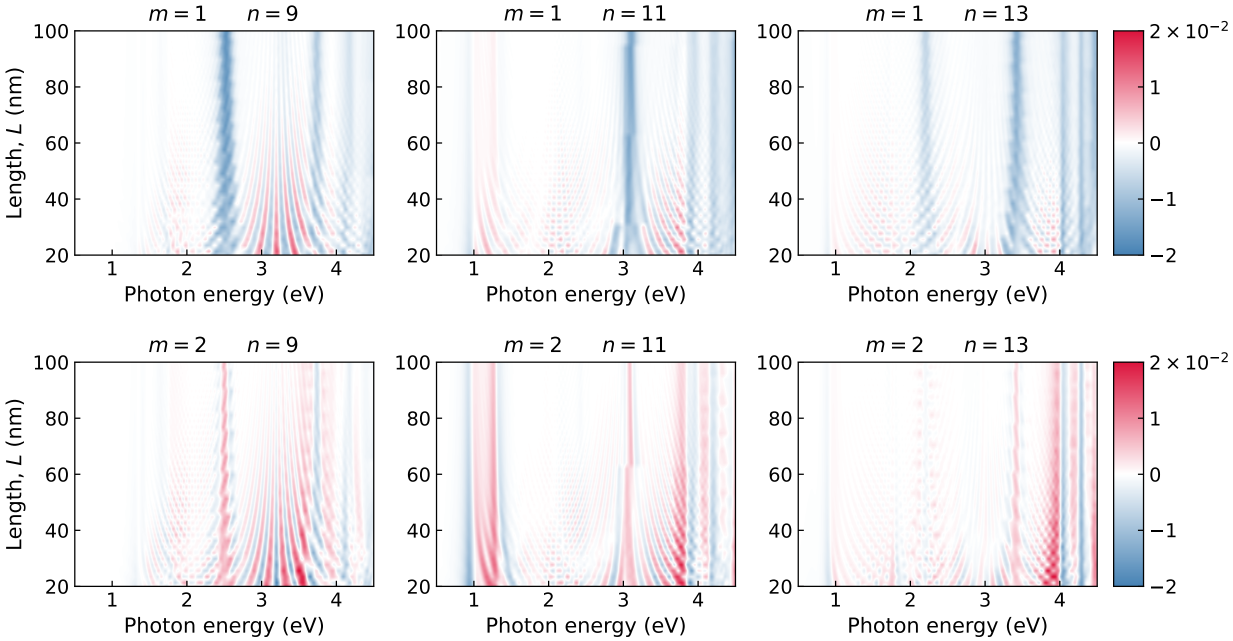

Figure S6: Dissymmetry factor spectra of chiral graphene nanostrips *vs* nanoribbon length L for $m = 1, 2$, $n = 9, 11, 13$, and $\chi = 1$.
