# Supplementary material for: Chiral Optical Properties of Möbius Graphene Nanostrips
Source: J Phys Chem Lett. 2023 May 4;14(19):4426–32. doi: 10.1021/acs.jpclett.3c00925 (PMC10201574; doi:10.1021/acs.jpclett.3c00925)
Supplement: Supplementary file 2 — jz3c00925_si_002.pdf [file jz3c00925_si_002.pdf]

jz-2023-009258.R1

Name: Peer Review Information for "Chiral Optical Properties of Möbius Graphene Nanostrips"

First Round of Reviewer Comments

Reviewer: 1

Comments to the Author

I think that, despite being only theoretical, the manuscript by Tepliakov et al. is interesting and appropriate for the Journal of Physical Chemistry Letters, since it presents a well written evaluation of chiral responses of twisted nanoribbons, by rationalizing the g dissymmetry ratio in terms of length (radius) and transverse dimension of the molecular systems, and also by taking into account overlapping cancellations of calculated bands.

I do not completely agree with the statement "the dissymmetry factors of twisted graphene nanostrips can reach 0.01, exceeding the dissymmetry factors of small chiral molecules by two to three orders of magnitude". Apart from lanthanide complexes, and just considering organic molecules, a couple of papers could be cited to this instance reporting g ratio of many molecules and evaluating in terms of fundamental constants a limiting value (10.1002/cptc.201800015 and 10.1002/cptc.202100222).

Since the authors claim that the study can be useful for nanostructures with chiral optical applications (for which matter I agree), it would be interesting if the Authors provided a comment/comparison between Moebius structures and belt geometries, as done for example for the ones studied in 10.1002/anie.202106992.

Reviewer: 2

Comments to the Author

Authors provide a theoretical analysis of the optical activity of Mobius graphene nanostrips, which were recently obtained in the experiment by the other group. To describe the Mobius geometry, they introduce a curvilinear coordinate space with cyclic boundary conditions. Analysis shows that dissymmetry factor (g-factor) can reach 10^-2 in small twisted graphene nanostrips, whereas it is on the scale of 10^-3 in the nanostructures with spherical geometry such as graphene quantum dots. G-factor is shown to depend on the number of twists in the strip that is in a good agreement with experimental observations of strong optical activity in twisted graphene nanoribbons. I recommend accepting this research paper for publication in JPC Letters as it is.

Author's Response to Peer Review Comments:

Dear Professor Editor,

Thank you very much for managing the review process for our manuscript. We are also thankful to the reviewers for their high appreciation of our work and valuable comments which further improved the quality of the manuscript. We are pleased to submit the revised version of our manuscript to The Journal of Physical Chemistry Letters.

We have fully addressed all the minor comments of Reviewer 1, as described in detail in a separate response file provided with the submission. We have also ensured that the revised manuscript complies with the style guidelines of the journal. Specifically, we have removed the section headings, provided a TOC image at the beginning of the document, and fixed the references style.

Thank you for your time and consideration. We look forward to your final decision on our manuscript.

Sincerely Yours,

Nikita Tepliakov

(on behalf of all authors)

Reply to Reviewer 1

Reviewer 1. *I think that, despite being only theoretical, the manuscript by Tepliakov et al. is interesting and appropriate for the Journal of Physical Chemistry Letters, since it presents a well written evaluation of chiral responses of twisted nanoribbons, by rationalizing the g dissymmetry ratio in terms of length (radius) and transverse dimension of the molecular systems, and also by taking into account overlapping cancellations of calculated bands.*

Authors. We thank Reviewer 1 for their careful reading and constructive comments on the manuscript, which have helped to improve it. Below we respond to each of their comments and describe any resulting revisions to the manuscript (the changes are highlighted by red).

I do not completely agree with the statement "the dissymmetry factors of twisted graphene nanostrips can reach 0.01, exceeding the dissymmetry factors of small chiral molecules by two to three orders of magnitude". Apart from lanthanide complexes, and just considering organic molecules, a couple of papers could be cited to this instance reporting g ratio of many molecules and evaluating in terms of fundamental constants a limiting value (10.1002/cptc.201800015 and 10.1002/cptc.202100222).

We thank the reviewer for this remark. Indeed, according to the literature, small chiral molecules typically have dissymmetry factors of the order of 10^{-3}–10^{-4}. We have corrected this sentence in the abstract of the manuscript:

"It is found that the dissymmetry factors of twisted graphene nanostrips can reach 0.01, exceeding the typical dissymmetry factors of small chiral molecules by one to two orders of magnitude."

We have also cited the papers suggested by the reviewer:

(44) Mazzeo, G.; Ghidinelli, S.; Ruzziconi, R.; Grandi, M.; Abbate, S.; Longhi, G. Circularly polarized luminescence of some [2]paracyclo[2](5, 8)quinoliphane derivatives with planar and central chirality. *ChemPhotoChem* **2022**, *6*, e202100222.

(45) Tanaka, H.; Inoue, Y.; Mori, T. Circularly polarized luminescence and circular dichroisms in small organic molecules: Correlation between excitation and emission dissymmetry factors. *ChemPhotoChem* **2018**, *2*, 386—402.

Since the authors claim that the study can be useful for nanostructures with chiral optical applications (for which matter I agree), it would be interesting if the Authors provided a comment/comparison between Moebius structures and belt geometries, as done for example for the ones studied in 10.1002/anie.202106992.

We have addressed this comment by adding the following sentence to the text of the manuscript:

"One sees that $g(\hbar\omega)$ can reach 10^{-2}, which is close to the experimental value for Möbius graphene nanobelts[31] and exceeds the typical values for small chiral molecules and quantum dots by one to two orders of magnitude.[19,46,47] Chiral molecular cylinders, morphologically similar to circular graphene nanostrips, can feature even larger dissymmetry factors of the order of 10^{-1}, though these values are highly sensitive to structural fluctuations.[48]"

The paper suggested by the reviewer has also been cited:

(48) Fukunaga, T. M.; Sawabe, C.; Matsuno, T.; Takeya, J.; Okamoto, T.; Isobe, H. Manipulations of chiroptical properties in belt-persistent cycloarylenes via desymmetrization with heteroatom doping. *Angew. Chem. Int. Ed.* **2021**, 60, 19097–19101.

Reply to Reviewer 2

Reviewer 2. *Authors provide a theoretical analysis of the optical activity of Mobius graphene nanostrips, which were recently obtained in the experiment by the other group. To describe the Mobius geometry, they introduce a curvilinear coordinate space with cyclic boundary conditions. Analysis shows that dissymmetry factor (g-factor) can reach* 10^{-2} *in small twisted graphene nanostrips, whereas it is on the scale of* 10^{-3} *in the nanostructures with spherical geometry such as graphene quantum dots. G-factor is shown to depend on the number of twists in the strip that is in a good agreement with experimental observations of strong optical activity in twisted graphene nanoribbons. I recommend accepting this research paper for publication in JPC Letters as it is.*

Authors. We thank Reviewer 2 for their critical assessment of our manuscript and high appreciation of our work.
